# Supplementary material for: Hypermethylation of the Gene Body in SRCIN1 Is Involved in Breast Cancer Cell Proliferation and Is a Potential Blood-Based Biomarker for Early Detection and a Poor Prognosis
Source: Biomolecules. 2024 May 12;14(5):571. doi: 10.3390/biom14050571 (PMC11118508; doi:10.3390/biom14050571)
Supplement: Supplementary file 1 [file biomolecules-14-00571-s001.zip › biomolecules-2982132-supplementary.pdf]

Hypermethylation of gene body in *SRCIN1* involved in breast cancer cell proliferation with potential as a blood-based biomarker for early detection and poor prognosis

Hsieh-Tsung Shen[1,2], Clilia Davis[3], Chih-Ming Su[4,5], Li-Min Liao[5], Hsiu-Ming Shih[1], Kuan-Der Lee[1], Chin-Sheng Hung[2,4,5,6]*, Muhamad Ansar[7*,] and Ruo-Kai Lin[1,2,3,7,8,9]*

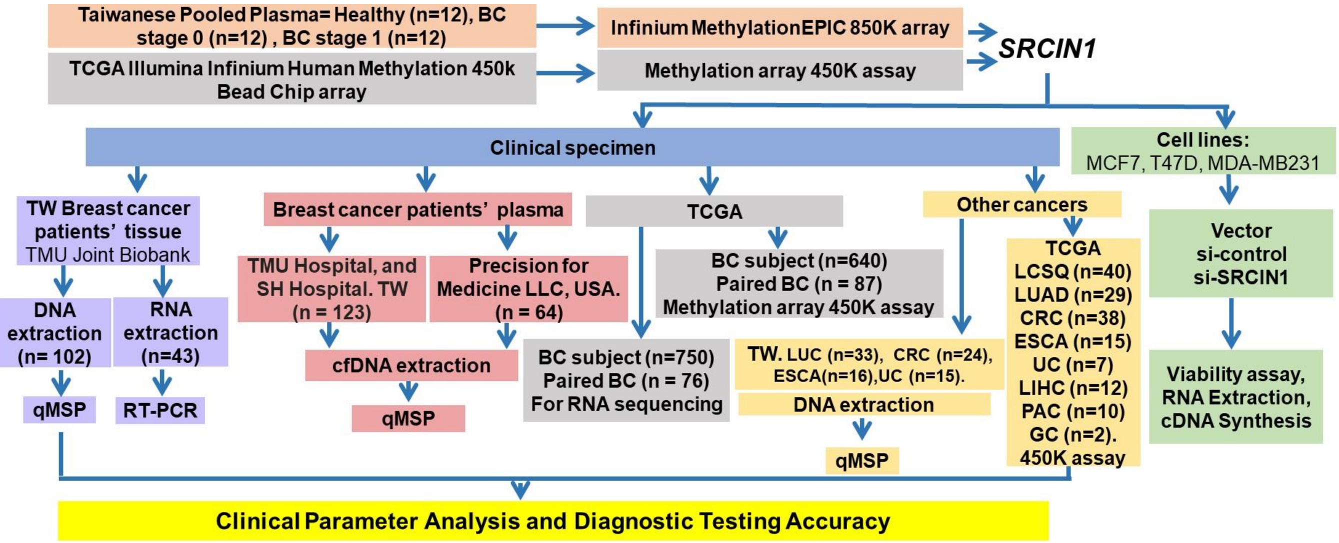

Figure S1. Flowchart of the study design, datasets, and specimens used. For each step, the sample types and number of samples used for the analyses are indicated. TW, Taiwan; TCGA, The Cancer Genome Atlas; BC, Breast Cancer; AN, adjacent normal; CRC, colorectal cancer; LCSQ, lung squamous cell carcinoma; lung adenocarcinoma; ESCA, esophageal cancer; UC, Uterine Cancer; LIHC, Liver Cancer; PAC, Pancreatic Cancer, cfDNA, circulating cell-free DNA; QMSP, quantitative methylation-specific PCR; qRT-PCR, quantitative reverse-transcription PCR, methylation450K array, Illumina Infinium HumanMethylation450 BeadChip array. methylation480K array, Illumina Infinium HumanMethylation850 BeadChip array.

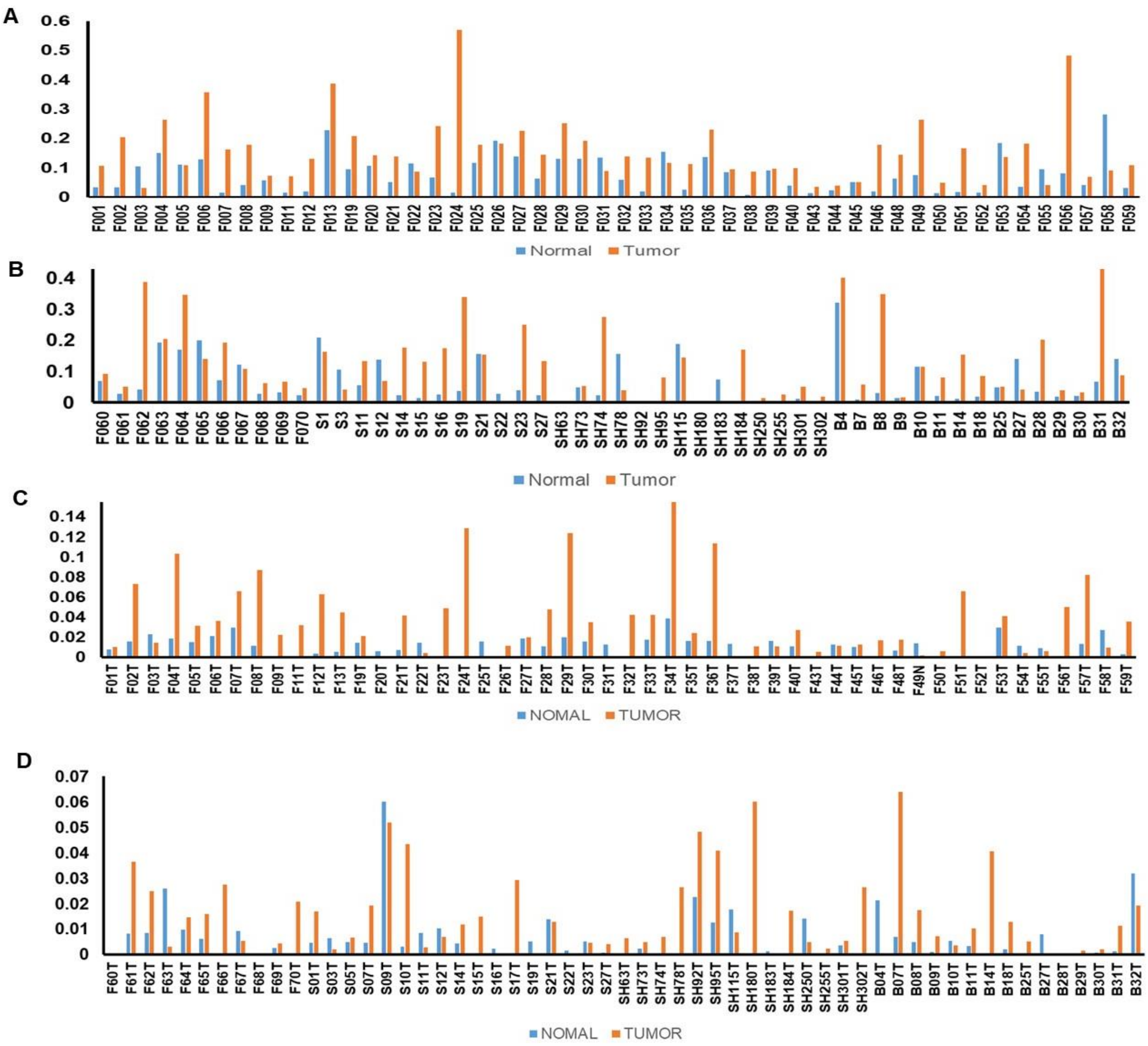

Figure S2. *SRCIN1* methylation level in Taiwanese breast cancer patients: **(A)(B)(C)(D)** Levels of methylated ***SRCIN1*** were determined using quantitative methylation-specific polymerase chain reaction (QMSP) in tumor tissue and adjacent normal tissue samples from 102 Taiwanese patients with breast cancer. ACTB was used as the internal control.

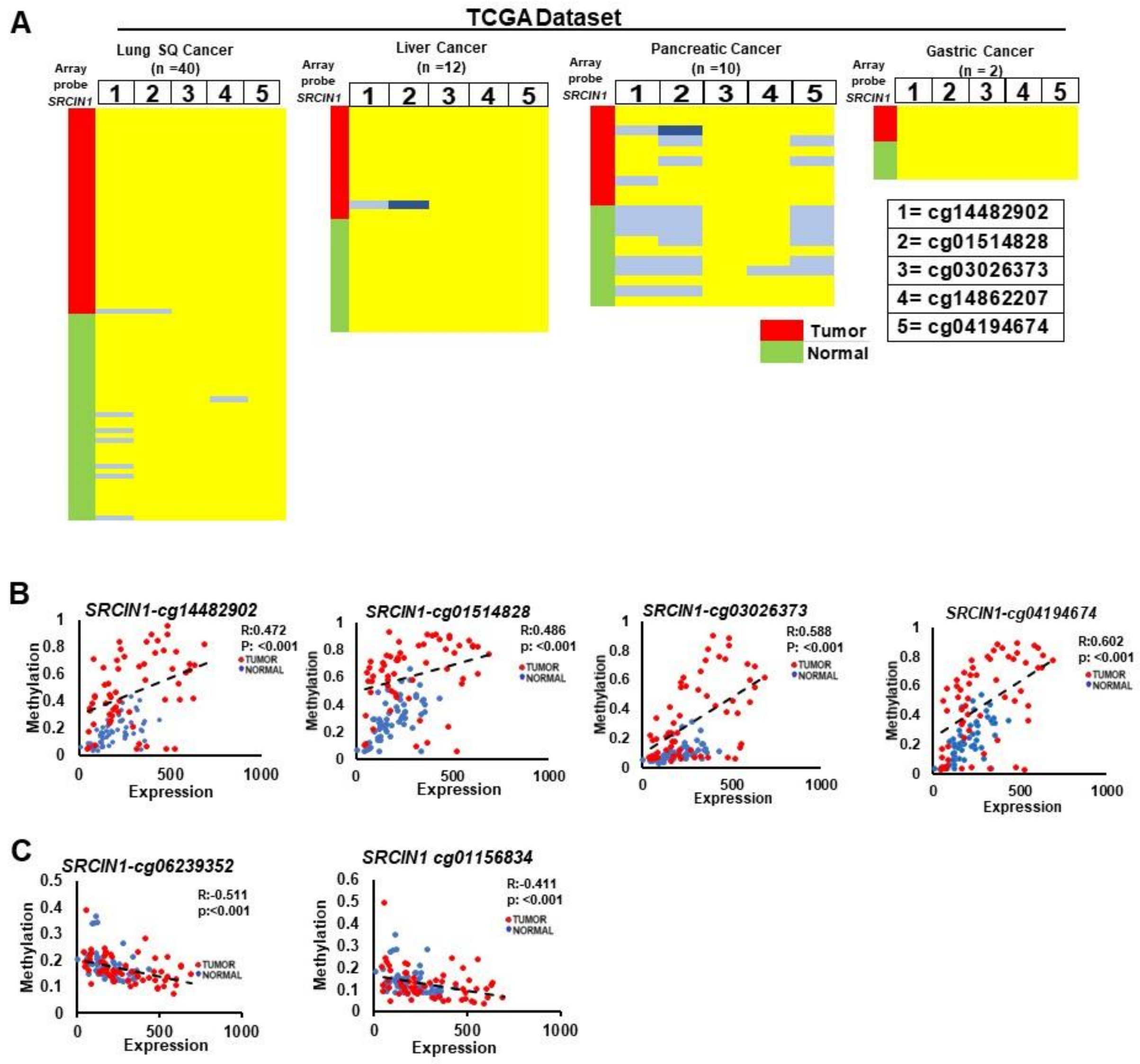

Figure S3. ***SRCIN1*** **DNA methylation in different cancers**. Differentially methylated CpG heatmap of *SRC1N1* **(A)** From TCGA dataset lung, liver cancer, pancreatic, and gastric cancer. Pearson correlation analysis of tissues between DNA methylation and RNA sequencing in 87 paired adjacent normal tissue samples and 87 paired breast cancer tissues from patients **(B)** Gene body **(C)** Promoter sites.

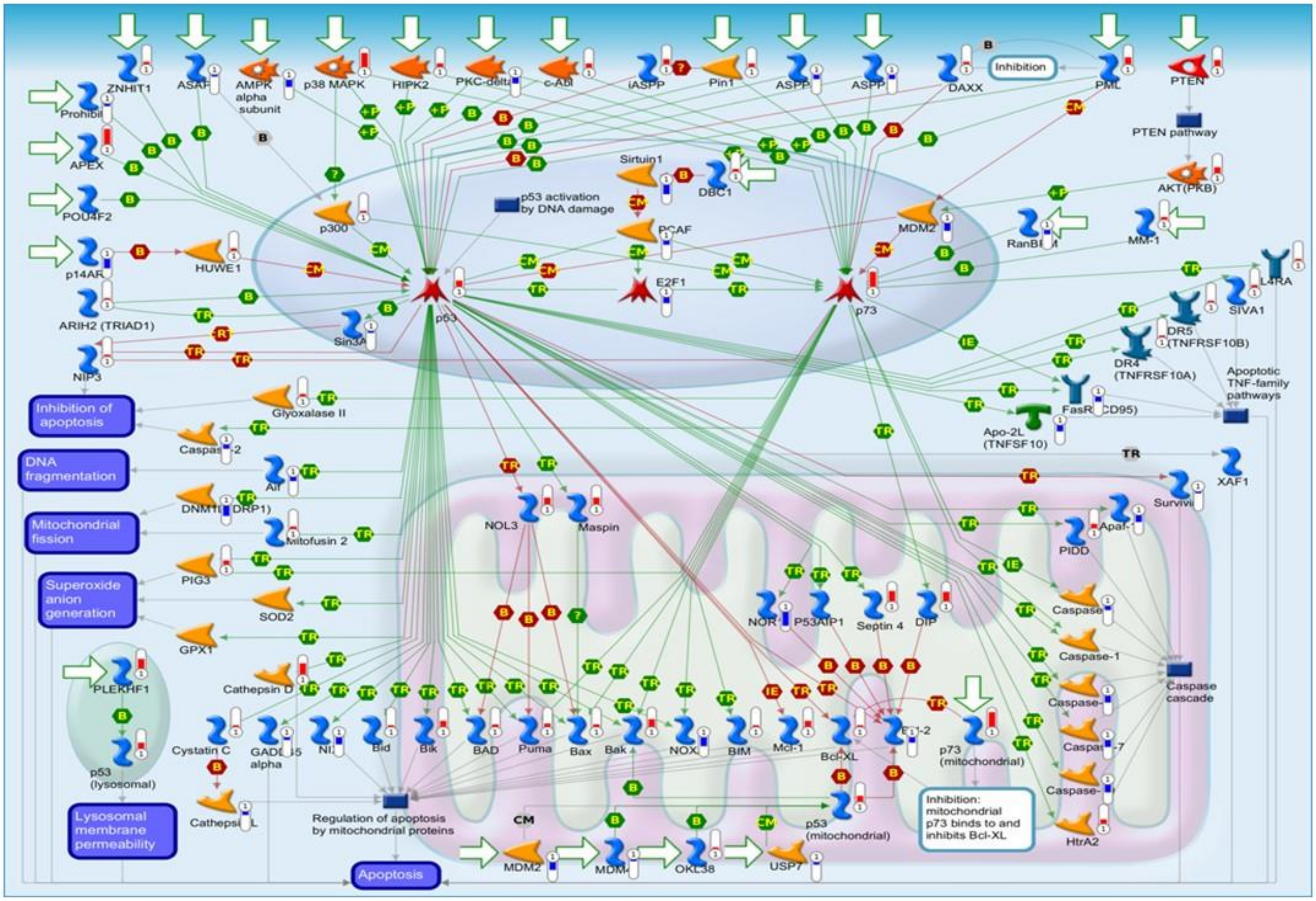

Figure S4. Network analysis of *SRCIN1* involved in apoptotic and survival pathways. MetaCore Metabolic Networks analysis found that *SRCIN1 was* involved in apoptotic and survival pathways after silencing *SRCIN1* in the MCF-7 cell line. RNA-seq data showed significant upregulation in p53, p73, p38, MPK, PML, iASPP, PTEN, APEX, Mcl-1, Bcl-W.

Table S1. List primer sequences and their reaction conditions used in the present study.

Gene	Primer	5′→3′sequences	Application
**ACTB**	Forward primer	TGGTGATGGAGGAGGTTTAGTAAGT	MSP-M
	Reverse primer	AACCAATAAAACCTACTCCTCCCTTAA	
	Probe	ACCACCACCCAACACACAATAACAAACACA	
**SRCIN1**	Forward primer	GTATGTTTATGGTGAGTTTTTGCGG	
	Reverse primer	CAAAAATACTATTCCTACAATTCGAA	
	Probe	TGTTTAGGTTGTTGATTTCGTGCGT	
**GAPDH**	Forward primer	AGCCACATCGCTCAGACAC	RT-PCR Real-time
	Reverse primer	GCCCAATACGACCAAATCC	
**SRCIN1**	Forward primer	AGCCCCGACAAAAGCAAA	
	Reverse primer	CCAAAGGAAGTCAATACAGGGATAG	
**CKS**	Forward primer	TGTTGTCGTGGATTTGGTGC	
	Reverse primer	TGCACCTCTCAGTGCCATTT	
**CDK2**	Forward primer	GGCACGTACGGAGTTGTGTA	
	Reverse primer	CATCCAGCAGCGTGTCCA	
**CDK6**	Forward primer	TCTGATTACCTGCTCCGCGA	
	Reverse primer	CAGAATCATTGCACCTGAGGG	
**CYCLIN A**	Forward primer	CGGTTCCTGGAAGTGGTTCA	
	Reverse primer	TGGGGCGATGTTTCTTCTCG	
**CYCLIN A2**	Forward primer	GGCGGTACTGAAGTCCGGG	
	Reverse primer	TTAAGGGGTGCAACCCGTCTC	
**SKP2**	Forward primer	TGGCTGAACATTTCAGTACTCTC	
	Reverse primer	AGCAAAGTCTGCAGGGCAAA	
**CRM1**	Forward primer	TGCCGATCTGAAAGCCACTT	
	Reverse primer	TGGGCTCATTTCGGTAGCAG	
**NCOA3**	Forward primer	GTGAGCTCCCAGGGTTTTCT	
	Reverse primer	CCACCCTCTGTTGTCGGAAG	
**P27KIP1**	Forward primer	ACCTGCAACCGACGATTCTT	
	Reverse primer	TTGGGGAACCGTCTGAAACA	

Table S2. *SRCIN1* methylation and expression level versus clinical parameters breast cancer patients; data were generated from TCGA datasets[a].

Characteristics	N[b] (%)	*SRCIN1* Methylation[c]			*p*-value	N (%)	*SRCIN1* mRNA Expression[d]			*p*-value
		Low (%)	Normal (%)	High (%)			Low (%)	Normal (%)	High (%)	
**Overall**	640					**755**				
**Tumor Stage**					0.673					0.048[c]
I and II	309 (70.5)	62 (20.1)	52 (16.8)	195 (63.1)		373 (71.3)	54 (14.5)	150 (40.2)	169 (45.3)	
III and IV	129 (29.5)	26 (20.2)	27 (20.9)	76 (58.9)		150 (28.7)	10 (6.7)	66 (44.0)	74 (49.3)	
**Tumor size**										0.004[c]
<65	449 (72.6)	91 (20.3)	83 (18.5)	275 (61.2)	0.293	542 (72.4)	66 (12.2)	245 (45.2)	231 (42.6)	
≥65	169 (27.4)	25 (14.8)	31 (18.3)	113 (66.9)		207 (27.6)	24 (11.6)	68 (32.9)	115 (55.6)	
**Histological Type**										0.000[c]
IDC	397 (71.3)	77 (19.4)	64 (16.1)	256 (64.5)	0.020[b]	170 (24.7)	4 (2.4)	53 (31.2)	113 (66.5)	
ILC	152 (27.3)	27 (17.8)	34 (22.4)	91 (59.9)		492 (71.6)	73 (14.8)	219 (44.5)	200 (40.7)	
Mixed type	8 (1.4)	5 (62.5)	0 (0.0)	3 (37.5)		25 (3.6)	1 (4.0)	17 (68.0)	7 (28.0)	
**Tumor size**					0.321					0.473
T0–T1	114 (25.9)	19 (16.7)	19 (16.7)	76 (66.7)		135 (25.6)	13 (9.6)	60 (44.4)	62 (45.9)	
T2–T4	327 (24.1)	69 (21.1)	61 (18.7)	197 (60.2)		392 (74.4)	52 (13.3)	158 (40.3)	182 (46.4)	
**Lymph nodes**					0.990					0.535
N0	198 (45.1)	40 (20.2)	35 (17.7)	123 (62.1)		237 (45.2)	25 (10.5)	99 (41.8)	113 (47.7)	
N1–N3	241 (54.9)	48 (19.9)	44 (18.3)	149 (61.8)		287 (54.8)	39 (13.6)	120 (41.8)	128 (44.6)	
**Race**					0.074					0.429
White	338 (78.4)	75 (86.2)	61 (18.0)	202 (59.8)		413 (79.9)	53 (12.8)	165 (40.0)	195 (47.2)	
American	30 (7.0)	5 (16.7)	9 (30.0)	16 (53.3)		72 (13.9)	7 (9.7)	32 (44.4)	33 (45.8)	
Asian	63 (14.6)	7 (11.1)	9 (14.3)	47 (74.6)		32 (6.2)	3 (9.4)	18 (56.3)	11 (34.4)	
**Menopause State**					0.695					0.124
Premenopause	7 (14.9)	1 (14.3)	3 (42.9)	3 (42.9)		121 (25.7)	18 (14.9)	55 (45.5)	48 (39.7)	
Perimenopause	5 (10.6)	2 (40.0)	0 (0.0)	3 (60.0)		16 (3.4)	3 (18.8)	9 (56.3)	4 (25.0)	
Postmenopause	35 (74.5)	6 (17.1)	11 (31.4)	18 (51.4)		333 (70.9)	37 (11.1)	129 (38.7)	167 (50.2)	

**Metastasis**					0.522					0.091
M0	376 (98.2)	74 (19.7)	70 (18.6)	232 (61.7)		449 (98.0)	59 (13.1)	191 (42.5)	199 (44.3)	
M1	7 (1.8)	1 (14.3)	0 (0.0)	6 (85.7)		9 (2.0)	1 (11.1)	7 (77.8)	1 (11.1)	
**Tumor Markers**										
Estrogen					0.081					
Negative	101 (22.9)	17 (16.8)	15 (14.9)	69 (68.3)		116 (22.1)	40 (34.5)	50 (43.1)	26 (22.4)	0.000[c]
Positive	340 (77.1)	71 (20.9)	66 (19.4)	203 (59.7)		408 (77.9)	24 (5.9)	166 (40.7)	218 (53.4)	
Progesterone					0.759					
Negative	141 (32.0)	28 (19.9)	23 (16.3)	90 (63.8)		164 (31.4)	43 (26.2)	69 (42.1)	52 (31.7)	0.000[c]
Positive	299 (68.0)	60 (20.1)	58 (19.4)	181 (60.5)		358 (68.6)	21 (5.9)	145 (40.5)	192 (53.6)	
HER2					0.609					
Negative	247 (84.9)	53 (21.5)	44 (17.8)	150 (60.7)		292 (64.6)	47 (16.1)	132 (45.2)	113 (38.7)	0.001[c]
Positive	44 (15.1)	7 (15.9)	11 (25.0)	26 (59.1)		56 (12.4)	3 (5.4)	16 (28.6)	37 (66.1)	
Equivoc						104 (23.0)	9 (8.7)	40 (38.5)	55 (52.9)	
**Subtypes**										
TNBC					0.374					
Negative	237 (81.7)	50 (21.1)	48 (20.3)	139 (58.6)		690 (92.0)	64 (9.3)	289 (41.9)	337 (48.8)	0.000[c]
Positive	53 (18.3)	10 (18.9)	7 (13.2)	36 (67.9)		60 (8.0)	26 (43.3)	25 (41.7)	9 (15.0)	
Luminal A					0.498					
Negative	97 (33.4)	17 (17.5)	18 (18.6)	62 (63.9)		116 (33.5)	29 (25.0)	41 (35.3)	46 (39.7)	0.000[c]
Positive	193 (66.6)	43 (22.3)	37 (19.2)	113 (58.5)		230 (66.5)	21	105 (9.1)	104 (45.7	
Luminal B					0.283					0.448
Negative	230 (79.3)	52 (22.6)	43 (18.7)	135 (58.7)		267 (77.2)	36 (13.5)	117 (43.8)	114 (42.7)	
Positive	60 (20.7)	8 (13.3)	12 (20.0)	40 (66.7)		79 (22.8)	14 (17.7)	29 (36.7)	36 (45.6)	

[a]These results were analyzed by Pearson's X2 test.
[b]For some categories, the number of samples (n) was lower than the overall number analyzed because clinical data were unavailable for those samples.
[c]*SRCIN1* methylation was considered hypermethylation when the *SRCIN1* methylation level was more than two in breast tumors compared to adjacent normal breast tissues.
[d]*SRCIN1* was considered high when the *SRCIN1* expression level was more than two in breast tumors compared to adjacent normal breast tissues.
